# Kidney Transplant Outcomes in Recipients Over the Age of 70

**DOI:** 10.7759/cureus.34021

**Published:** 2023-01-20

**Authors:** Jaya Mehta, Okonkwo Ndubueze, Daniel Tatum, Hoonbae Jeon, Anil Paramesh, Mary Killackey, Adarsh Vijay

**Affiliations:** 1 Transplant Surgery, Tulane University School of Medicine, New Orleans, USA

**Keywords:** transplant survival, immunosuppression, outcomes, kidney transplant, elderly

## Abstract

Background: Patients older than 70 years are the fastest-growing age group of patients requiring renal replacement therapy. This has resulted in a corresponding increase in the number of elderly transplant recipients. We hypothesized that graft survival in this population would be comparable to that seen in the literature on kidney transplant recipients under 70 years of age.

Methods: This was a retrospective, single-center review of outcomes of kidney transplant recipients aged ≥70 years. Patients were dichotomized based on whether their allograft originated from a living or deceased donor.

Results: A total of 59 recipients aged ≥70 years underwent kidney transplantation. Of these, five (8.5%) were lost to follow-up within the first year post transplant and excluded from the analysis. History of cerebrovascular accident (p = 0.003), coronary artery disease (p = 0.03), postoperative return to the operating room (p = 0.03), and readmission within one year of transplant were predictive of graft loss (p = 0.003). Overall graft survival in our cohort declined from 92.6% at one year to 53.8% at five years. Death-censored graft survival was 100% at one year and decreased to 80.8% at five years. There were no differences seen in patient, graft, or death-censored graft survival based on donor type.

Conclusions: Kidney transplant patients over 70 years, as seen in our cohort, had good short-term outcomes. Graft survival is similar to rates seen in younger cohorts but the decline in this rate over time is steeper in the older age group, possibly due to decreased patient survival. These findings could be validated further in larger multi-center studies.

## Introduction

The scientific and technological advances over the last century have supplemented increasing public health awareness to increase longevity. This has slowly led to a rising population of older adults that are living significantly longer than their predecessors [1]. It is estimated that by 2060, the median age of the United States population will be 43 (up from 38 today) and that the percentage of the population above 65+ years will surpass the percentage that is below 18 [2]. This will significantly affect the health system as it will bring along with it an increased burden of the disease processes that accompany the aging body. One such age-associated burden is a decline in renal function. Chronic kidney disease (CKD) is prevalent in 44% of people above the age of 70 [3], and this number has been increasing due to an increased incidence of type 2 diabetes mellitus (DM), and hypertension (HTN), which lend themselves to the development of CKD. Together, the number of elderly patients requiring renal replacement therapy has resulted in their increasing representation on kidney transplant wait lists [3,4]. About half of these patients die while on the waitlist in the United States [5].

Previous studies have shown improved quality of life and a 41% reduction in mortality risk in elderly patients who received a kidney transplant when compared to dialysis-dependent wait-listed patients [3-6]. Most studies in the past that have addressed outcomes in elderly kidney transplant recipients have pooled all patients above the age of 65 into a single group [6-9]. Significant decline is noted in kidney function between the ages of 65 and 70 [10]. Thus, there remains a need to address the clinical outcomes in 70+-year-old recipients separately to ascertain the transplant benefit to this sub-population. In this study, we investigated the short- and long-term clinical outcomes of kidney transplant recipients aged 70 years or greater at the time of their transplant.

## Materials and methods

Study population

**Figure 1:**
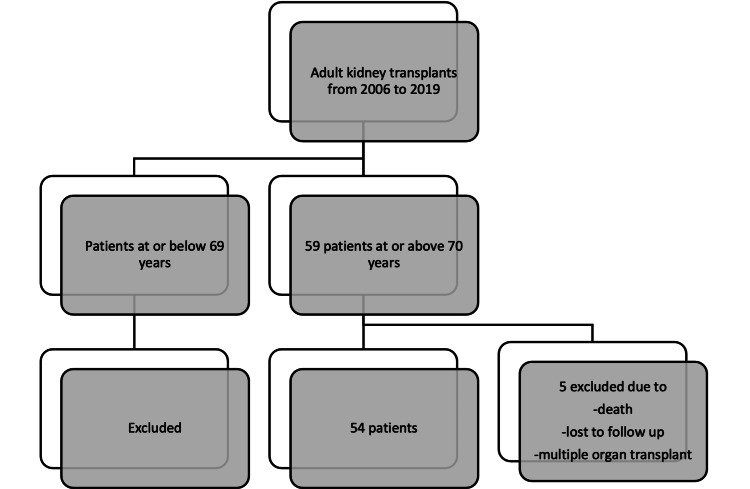
Flow diagram representing exclusion criteria for cohort

This was a single-center, retrospective cohort study approved by the institutional review board (IRB) of Tulane University, New Orleans, Louisiana, United States (approval number: 2022-1518) and a waiver of informed consent was granted by the IRB. All kidney transplant recipients who were aged 70 years or more at the time of their transplant at Tulane Medical Center, New Orleans, Louisiana between January 1, 2006, and December 31, 2019, were identified using data submitted to the United Network for Organ Sharing (UNOS) [11]. Patients were excluded if lost to follow-up within the first year post transplant (Figure 1)

Data collection and study elements

Donor and recipient characteristics were identified using the site-specific UNOS database [11]. Recipient data were collected from hospital electronic medical records. Data collection was independently done by two authors (JM and ON) and rechecked by a senior author (AV). The variables examined included donor characteristics such as age, sex, race, blood type, BMI, kidney donor profile index (KDPI) [12], terminal creatinine of donor prior to donation, preservation/cold ischemic time (time when the organ is preserved in a hypothermic state prior to transplantation into the recipient), and donor status (donation after brain death (DBD)/donation after cardiac death (DCD)).

Demographic and clinical outcome descriptors of transplant recipients, such as age, sex, race, BMI, blood type, education level, and waiting list time (waiting time is calculated from the date when the patient starts dialysis or the date the transplant center listed the patient, whichever comes first), comorbidities, polypharmacy (defined as the use of five or more drugs before transplant), functional status at listing (defined as the performance of activities of daily living), anticoagulation/antiplatelet therapy use before transplant, and need for midodrine pre-transplant for hypotension (midodrine is used in dialysis patients to treat hypotension) were surveyed.

Operative and perioperative variables collected included warm ischemic time (WIT), hospital length of stay (LOS), 30-day hospital readmission, delayed graft function (DGF, defined as requiring dialysis within seven days post transplant), and postoperative complications including atrial fibrillation, hypotension, myocardial infarction, and return to the operating room. Immunologic characteristics included ABO incompatibility, human leukocyte antigen (HLA) mismatches, donor-recipient (DR) mismatch, and induction and maintenance immunosuppression regimen used.

The primary outcomes of interest were patient survival, graft survival, and death-censored graft survival at one, three, and five years following the kidney transplant. Graft failure was defined as a return to dialysis, repeat kidney transplant, or death with a functioning graft. Death-censored graft survival was used to estimate the probability of graft loss only. In the event of death with a functioning graft, the date of death is taken as the date of the last follow-up. The fatal event is thereby handled as a case lost to follow-up, not as a graft failure. Secondary outcomes of interest included perioperative complications, hospital LOS, re-admissions, and incidence of rejection, infection, or cancer in the follow-up period.

Statistical analysis

Patients were dichotomized by donor type (living vs deceased). Descriptive statistics were used to examine the cohorts. An examination of factors affecting one-year and three-year graft survival was also performed with patients categorized by graft survival or loss at the specified time frame. Categorical values were reported as frequencies, represented as n (%), and examined by Chi-square analysis. Continuous variables were reported as median (interquartile range (IQR)25 - IQR75)) and examined by the Mann-Whitney U test. The level of statistical significance was set at p < 0.05. All statistical analyses were performed using IBM SPSS Statistics for Windows, Version 28.0 (Released 2021; IBM Corp., Armonk, New York, United States).

## Results

A total of 54 kidney transplant recipients were included in the analysis, of which 17 (31.5%) had living donors and 37 (68.5%) deceased donors. The overall cohort had a mean age of 72 years and a BMI of 27.1, was predominantly of white race (64.8%), and had a high school education or lower (64.8%) (Table 1)

**Table 1:** Description of total patient cohort and living donor recipient and deceased donor recipient subgroups All continuous variables are reported as median (IQR25 – IQR75). Categorical values are frequencies reported as n (%).

Parameter	Total (n = 54)	Living donor (n = 17, 31.5%)	Deceased donor (n = 37, 68.5%)	P
Age, years	72 (70.8-74.3)	73 (71-76.5)	72 (70-73.5)	0.30
BMI at listing	27.6 (25.1-31.3)	29.3 (26-31.1)	27.1 (24.9-31.5)	.36
Waitlist time, days	512.5 (137.5-1165.5)	119 (70-280.5)	737 (258-1580.5)	<0.001
Sex				0.03
Male	33 (61.1)	14 (82.4)	19 (51.4)	
Female	21 (38.9)	3 (17.6)	18 (48.6)	
Race/ethnicity				0.75
White	35 (64.8)	12 (70.6)	23 (62.2)	
African American/Black	16 (29.6)	4 (23.5)	12 (32.4)	
Asian	1 (1.9)	0 (0.0)	1 (2.7)	
Hispanic	2 (3.7)	1 (5.9)	1 (2.7)	
Education level				0.22
High school or below	35 (64.8)	9 (52.9)	26 (70.3)	
College and more	19 (35.2)	8 (47.1)	11 (29.7)	
Blood type				0.56
A	25 (46.3)	9 (52.9)	16 (43.2)	
B	3 (5.6)	0 (0.0)	3 (8.1)	
AB	1 (1.9)	0 (0.0)	1 (2.7)	
O	25 (46.3)	8 (47.1)	17 (45.9)	
Comorbidities				
Diabetes mellitus	22 (40.7)	5 (29.4)	17 (45.9)	0.25
Hypertension	53 (98.1)	16 (94.1)	37 (100.0)	0.14
Coronary artery disease	15 (27.8)	7 (41.2)	8 (21.6)	0.14
Cerebrovascular accient	5 (9.3)	1 (5.9)	4 (10.8)	0.56
Polypharmacy at transplant	47 (87.0)	14 (82.4)	33 (89.2)	0.49
Functional status at listing				0.05
60% - Requires occasional assistance but is able to care for needs	2 (3.7)	1 (5.9)	1 (2.7)	
70% - Cares for self: unable to carry on normal activity or active work	12 (22.2)	3 (17.6)	9 (24.3)	
80% - Normal activity with effort: some symptoms of disease	15 (27.8)	1 (5.9)	14 (37.8)	
90% - Able to carry on normal activity: minor symptoms of disease	25 (46.3)	12 (70.6)	13 (35.1)	
Anticoagulation/antiplatelet before transplant	27 (50.0)	11 (64.7)	16 (43.2)	0.14
Minodrine use before transplant	1 (1.9)	0 (0.0)	1 (2.7)	0.49

There were no differences between groups in any of these demographic factors. There were more male recipients than female recipients, and a higher proportion of male patients received living donors than deceased donor organs (p = 0.03). The majority of the recipients had blood group A or O, with the remainder being either AB or B type, but these proportions did not differ between cohorts. The most common comorbidity present at the time of listing was HTN, followed by DM, coronary artery disease (CAD), and a history of cerebrovascular accident (CVA); these proportions did not differ between the cohorts. Most (87%) recipients were taking five or more pharmacological agents at the time of transplant, and half of the total cohort was on anticoagulation/antiplatelet therapy. Most recipients (46.3%) were documented as having a functional status at the listing of 90%, while only 3.7% had a functional status below 70%. The distribution of the degree of functional ability did not differ between cohorts. Living donor recipients had a significantly shorter waiting list time compared to deceased donor recipients (p <0.001). Donor characteristics are detailed in Table 2.

**Table 2:** Donor characteristics All continuous variables are reported as median (IQR25 – IQR75). Categorical values are frequencies reported as n (%).

Parameter	Total	Living donor	Deceased donor	P
Age, years	45.5 (28.3-51.3)	48 (45-51)	42 (24.5-55.2)	0.10
Sex				0.21
Male	29 (53.7)	7 (41.2)	22 (59.5)	
Female	25 (46.3)	10 (58.8)	15 (40.5)	
Race/Ethnicity				0.13
White	34 (63.0)	14 (82.4)	20 (54.1)	
African American/Black	15 (27.8)	2 (11.8)	13 (35.1)	
Hispanic	5 (9.3)	1 (5.9)	4 (10.8)	
Donor BMI (Kg/m2)	54	27.8 (23.3-31.7)	26.3 (23-31.4)	0.47
Kidney Donor Profile Index	0.59 (0.28-0.64)	-	0.59 (0.28-0.64)	-
Cold ischemic time, min	54	0.61 (0.5-1.0)	16.8 (13.9-22.0)	<0.001
Kidney pump used	8 (14.8)	0 (0.0)	8 (21.6)	0.04
Donor Term Creatinine (mg/dl)	0.92 (0.70-1.25)	0.92 (0.70-1.25)	-	-

The donor cohort was of median age of 45.5 years, median BMI of 26.8, majority male (53.7%), and majority white race (63.0%). The KDPI for the deceased donor group was 59%. The cold ischemia time was higher for the deceased donor group at 16.8 hours vs 0.6 hours in the living donor group (p < 0.001). Kidneys were pumped prior to transplantation in 21.6% of the deceased donor cases.

Table 3 details patient outcomes and immunosuppression regimens. Basiliximab was the preferred induction immunosuppressive agent in the cohort and was utilized in 50% of patients, followed by alemtuzumab (37%) and Thymoglobulin (7.4%). The proportions of patients receiving these regimens did not differ between groups. Most (81.5%) were on calcineurin inhibitors and mycophenolate for maintenance immunosuppression. Mycophenolate was discontinued in 18.5% of recipients owing to mycophenolate intolerance/side effects. There were no differences in human leukocyte antigen - DR isotype (HLA-DR) mismatch based on donor type, with 50% overall with a mismatch of 1, 24.1% with 2, and 25.9% with 0. Patient outcomes based on donor type were compared (Table 3).

**Table 3:** Immunosuppression, matching, and perioperative concerns AMR: antibody-mediated rejection; ACR: acute cellular rejection; CNI: calcineurin inhibitor; MMF: mycophenolate mofetil; HLR-DR: human leukocyte antigen-DR isotype)

Parameter	Total (n = 54)	Living donor (n = 17)	Deceased donor (n = 37)	P-value
Length of stay, days	6 (4.8-8.3)	7 (5-8.5)	6 (4-8)	0.23
Delayed graft failure	11 (20.4)	2 (11.8)	9 (24.3)	0.29
Infection				
<1 year	27 (51.9)	8 (50.0)	19 (52.8)	0.85
1-10 years	18 (39.1)	3 (20.0)	15 (48.4)	
Cancer				
<1 year	2 (3.8)	1 (6.3)	1 (2.8)	0.55
1-10 years	15 (33.3)	6 (40.0)	9 (30.0)	0.50
Rejection				
<1-year AMR/ACR	5 (9.6)	0 (0.0)	5 (14.3)	0.10
After 1-year AMR/ACR	0 (0.0)	0 (0.0)	0 (0.0)	
Post-op complications				
Atrial fibrillation	4 (8.0)	2 (12.5)	2 (5.9)	0.42
Hypotension	2 (4.0)	1 (6.3)	1 (2.9)	0.58
Myocardial Infarction	1 (2.0)	1 (6.3)	0 (0.0)	0.14
Return to the operating room	7 (14.0)	3 (18.8)	4 (11.8)	0.51
30-day readmission	14 (26.9)	5 (29.4)	9 (25.7)	0.78
Readmission 30 days to 1 year	30 (57.7)	7 (43.8)	23 (63.9)	0.18
Immunosuppression				
Induction agent				
None	3 (5.6)	2 (11.8)	1 (2.7)	0.11
Campath	20 (37.0)	5 (29.4)	15 (40.5)	
Simulect	27 (50.0)	7 (41.2)	20 (54.1)	
Thymoglobulin	4 (7.4)	3 (17.6)	1 (2.7)	
Immunosuppression				0.11
CNI + MMF	44 (81.5)	16 (94.1)	28 (75.7)	
CNI	10 (18.5)	1 (5.9)	9 (24.3)	
HLA-DR mismatch				0.82
0	14 (25.9)	4 (23.5)	10 (27.0)	
1	27 (50.0)	8 (47.1)	19 (51.4)	
2	13 (24.1)	5 (29.4)	8 (21.6)	

Of the postoperative complications examined, atrial fibrillation was seen in 8% of the cohort, hypotension in 4%, and myocardial infarction in 2%. A total of seven patients (14% of cases) required a return to the operating room within that hospital stay, 26.9% were readmitted to a hospital within 30 days of the transplant, and 57.7% had at least one hospital readmission after 30 days but before one year after transplant. There were no differences in readmission rates based on donor organ type. DGF was observed at a higher rate in the deceased donor group (24.3%) when compared to the living donor group (11.8%), but this difference was not significant (p = 0.29). The median LOS postoperatively was six days and was comparable between both living donor and deceased donor groups.

The incidence of infection in the first year post transplant was 51.9%, and this decreased to 39.1% after one year. There were no differences in the incidence of infectious complications in the first year post transplant between deceased and living donor groups. The most frequently occurring pathogen types were bacterial, seen in 59.3% of patients (through common UTIs), and viral, seen in 48.2% of patients (cytomegalovirus (CMV), BK virus (BKV)). Cancer was seen in 3.8% of the total cohort within one year of transplant, and in 33.3% after one year. The incidence of rejection in the first year post transplant was 9.6%. Acute cellular rejection (ACR) was the most common type of rejection (three patients), followed by combined ACR and antibody-mediated rejection (AMR) in one patient.

**Figure 2:**
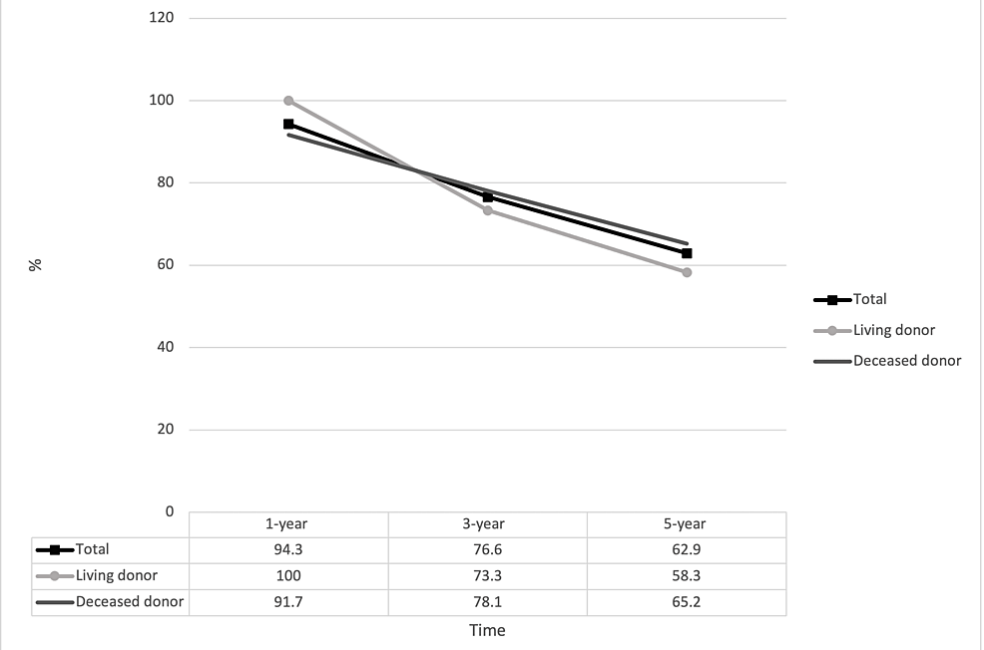
Patient survival in kidney transplant in patients aged ≥ 70 years at one year, three years, and five years post transplant. No differences were observed between living and deceased donor recipients p-values are displayed in their respective columns

**Figure 3:**
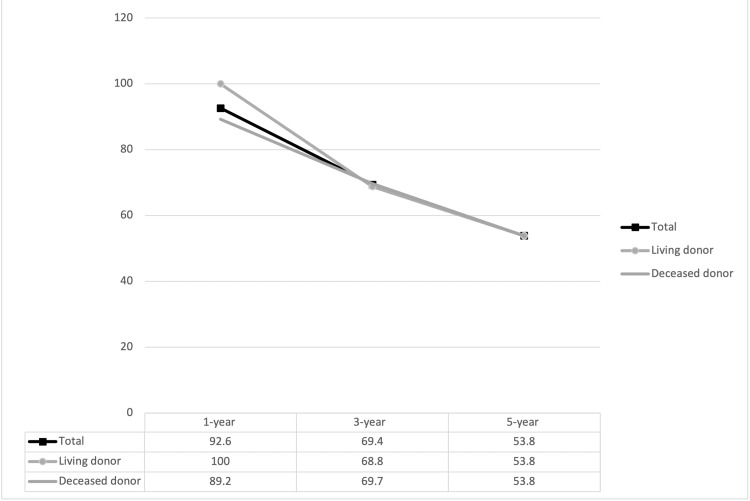
Graft survival in kidney transplant in patients aged ≥ 70 years at one year, three years, and five years post transplant. No differences were observed between living and deceased donor recipients. p-values are displayed in their respective columns

**Figure 4:**
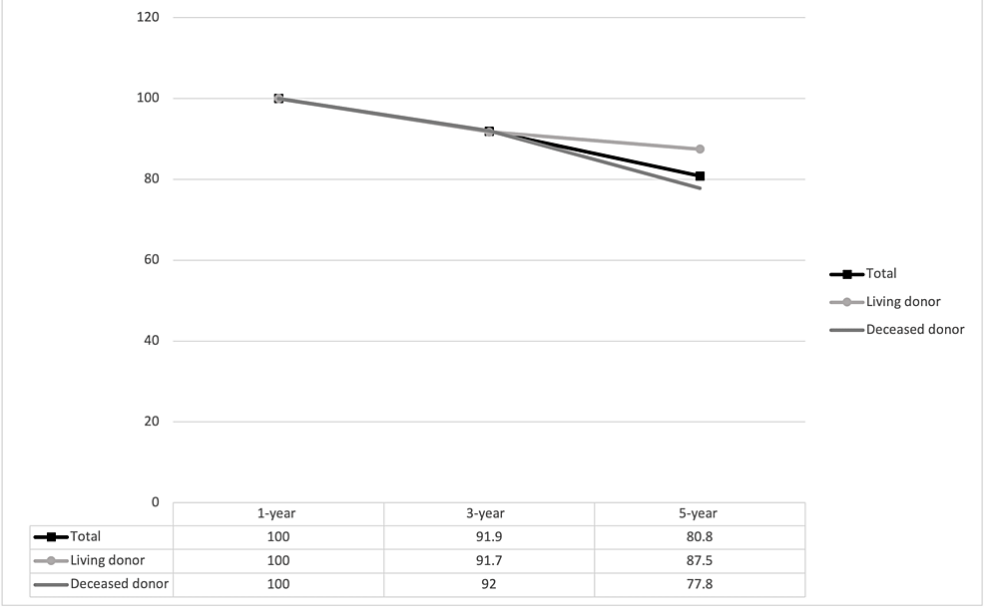
Death-censored graft survival in kidney transplant in patients aged ≥ 70 years at one year, three years, and five years post transplant. No differences were observed between living and deceased donor recipients p-values are displayed in their respective columns

Rates of one, three, and five-year graft survival, death-censored graft survival, and patient survival are presented in Figures 2, 3, 4. The one, three, and five-year rates of patient survival, graft survival, and death-censored graft survival decreased over each time period assessed.

Factors impacting graft survival were then examined. Specifically, recipient characteristics, comorbidities, peri-operative complications, incidence of rejection, cancer, and infections were correlated with one and three-year graft survival (Table 4).

**Table 4:** Factors affecting one and three-year graft survival in kidney transplant in patients aged ≥ 70 years

	1-year graft status				3-year graft status			
Parameter	Survival (n = 50)	Loss (n = 4)		P	Survival (n = 34)	Loss (n = 15)		P
Age, years	73.0 (3.4)	73.3 (1.9)		0.38	72.4 (2.6)	74.3 (3.9)		0.12
BMI at listing	28.1 (5.3)	27.5 (4.4)		0.59	28.4 (4.9)	27.3 (5.7)		0.69
Sex				0.55				0.43
Male	30 (60.0)	3 (75.0)			21 (61.8)	11 (73.3)		
Female	20 (40.0)	1 (25.0)			13 (38.2)	4 (26.7)		
Race/Ethnicity				0.14				0.13
White	33 (66.0)	2 (50.0)			24 (70.6)	8 (53.3)		
African American/Black	15 (30.0)	1 (25.0)			9 (26.5)	5 (33.3)		
Asian	1 (2.0)	0			1 (2.9)	0		
Hispanic	1 (2.0)	1 (25.0)			0	2 (13.3)		
Comorbidities								
Diabetes mellitus	21 (42.0)	1 (25.0)		0.51	14 (41.2)	7 (46.7)		0.72
Hypertension	49 (98.0)	4 (100.0)		0.78	33 (97.1)	15 (100.0)		0.50
Coronary artery disease	14 (28.0)	1 (25.0)		0.90	6 (17.6)	7 (46.7)		0.03
Cerebrovascular accident	3 (6.0)	2 (50.0)		0.003	1 (2.9)	3 (20.0)		0.04
Polypharmacy	44 (88.0)	3 (75.0)		0.46	30 (88.2)	13 (86.7)		0.88
Minodrine use before transplant	1 (2.0)	0		0.78	1 (2.9)	0		0.50
Anticoagulation/antiplatelet before transplant	24 (48.0)	3 (75.0)		0.30	16 (47.1)	10 (66.7)		0.21
Rejection < 1 year	4 (8.2)	1 (33.3)		0.15	2 (6.1)	3 (21.4)		0.12
Infection < 1 year	24 (50.0)	3 (75.0)		0.34	14 (42.4)	9 (64.3)		0.17
Cancer < 1 year	1 (2.0)	1 (33.3)		0.006	0	2 (15.4)		0.02
Postoperative complications								
Atrial fibrillation	4 (8.7)	0		0.54	4 (12.9)	0		0.16
Hypotension	2 (4.3)	0		0.67	2 (6.5)	0		0.33
Myocardial Infarction	1 (2.2)	0		0.77	1 (3.2)	0		0.50
Return to operating room	5 (10.9)	2 (50.0)		0.03	4 (12.9)	3 (21.4)		0.47
30day readmission	13 (27.1)	1 (25.0)		0.93	8 (25.0)	3 (20.0)		0.71
Readmission 30 days to 1 year	26 (54.2)	4 (100.0)		0.08	13 (40.6)	13 (86.7)		0.003
Delayed graft failure	8 (16.0)	3 (75.0)		0.005	5 (14.7)	5 (33.3)		0.14
Length of stay, days	8.6 (11.3)	9.3 (9.2)		0.62	9.8 (13.5)	6.9 (4.7)		0.52

Recipient demographics including age, sex, race, education level, blood type, functional status, BMI, and polypharmacy at the listing time did affect one or three-year graft survival in our study population.

Patients with a history of cerebrovascular disease were observed to have a higher (50% vs 6%) incidence of graft loss at one year (p = 0.003) and at three years post transplant (p = 0.04). History of CAD also affected graft function, with 46.7% of those with graft loss at three years having a history of CAD (p = 0.03). Out of those with graft failure at one year, 75% had at least one episode of infection and 33.3% had a diagnosis of cancer within the first year post transplant. Cancer within one year after transplant adversely affected 1 and 3-year graft survival with an incidence of 33.3% (p = 0.006) and 15.4% (p = 0.02) in patients who incurred graft loss.

Post-operative incidence of atrial fibrillation, hypotension, myocardial infarction, and length of initial hospital stay did not adversely affect one-year or three-year graft survival. Patients that required a return to the operating room after their transplant faired poorer than those who did not- 50% of patients who had graft failure at one year were taken back to the operating room postoperatively, compared with 10.9% with a functioning graft (p = 0.03). Readmission after 30 days but within the first year post transplant was seen in 86.7% of patients with a failed graft at three years whereas only 40.6% in those with surviving grafts (p= 0.003). DGF was seen in 75% of those with a failed graft within the first year (p = 0.005).

## Discussion

Kidney transplantation is the preferred modality for the treatment of end-stage renal disease (ESRD) owing to improved survival benefits and quality of life [13,14]. However, this benefit diminishes with increasing recipient age [13]. Owing to the variability in age cut-off used in studies that have examined outcomes in elderly kidney transplant recipients, it remains unclear at what age the predicted transplant advantage is lost. The present study aimed to describe outcomes after kidney transplantation in recipients 70 years and older.

Elderly waitlisted dialysis-dependent candidates have a 50% mortality rate while waiting for a kidney transplant, and this has been on the rise since 2019 [15,16]. There have been only a handful of studies that have looked at outcomes in transplant patients over the age of 70 years [14,17-19]. Rao et al. in 2007 reported a 41% lower overall risk of death in transplanted candidates when compared to their counterparts who remained on the waitlist [20]. Heldal et al in 2010 reported lower mortality in transplanted 70+-year-old dialysis-dependent recipients in comparison to those that stayed on dialysis [18]. In our study cohort, we witnessed excellent one-year graft survival of 92.6% and patient survival of 94.3%, which is comparable to other reported literature [13,16-18].

There is limited data regarding transplant outcomes in this subpopulation of elderly patients compared to their younger counterparts. In 2004, Fabrizii et al. reported a higher five-year patient survival amongst kidney transplant recipients in the age group of 50-59 years when compared to patients older than 60 [21]. Huang and colleagues at UCLA in 2010 stratified over 31,000 patients by age and reported two-year graft survival at 85% for the 60-69 years group, 81% for the 70-79 years group, and 69% for the 80 years and older group [22]. More recently, in 2021, Doucet et al. compared outcomes in transplant recipients between the ages of 18 and 69 years and those 70 years and above. They found that patients in the 18-69 years group who received a deceased donor organ had a graft survival of 93% at one year, 88% at three years, and 82% at five years, whereas patients aged 70 years and above who received a deceased donor organ had a graft survival of 93% at one year, 80% at three years, and 74% at five years [23]. Lemoine et al., in 2019, reported patient survival of 68.1% at five years [19]. Boesmueller and colleagues in 2011 reported similar five-year patient survival at 67%. In this study, the above 70 years group had a 27% lower graft survival rate at five years in comparison to the younger age group [18]. Our study witnessed the same trend of decreased graft and patient survival at the three-year and five-year follow-ups. Three-year and five-year patient survivals in our cohort were 76.6% and 62.9%, respectively, and three-year and five-year graft survivals were 69.4% and 53.8%, respectively. One-year, three-year, and five-year patient and graft survivals in living donor recipients were comparable to deceased donor recipients in our study cohort.

As of 2021, there are over 90,000 patients in the United States waiting for a kidney transplant, with 13 patients dying every day on the waitlist [16]. The one, three, and five-year death-censored graft survival in our study was 100%, 91.9%, and 80.8%, respectively (Figure 4). Death with a functioning allograft in the older recipient transplant population can rationale the stark difference between long-term graft survival and death-censored graft survival rates. This observation brings forward the concern of whether elderly transplant recipients are denying the transplantation chances of younger recipients who are expected to have a greater life expectancy. Aging can be a reliable proxy for stronger predictors such as functional status, physiologic organ reserve, or comorbidity; however, an age cut-off for the rationale to not transplant can raise several ethical concerns.

Studies have reported higher perioperative surgical complications in the elderly [24]. From our cohort, 14% needed to be taken back to the operating room and 26.9% had a readmission within 30 days of the procedure; 57.7% of recipients had a hospital re-admission in the first year post transplant. Post-transplant incidence of infection in our study, measured as any infection within one year after transplant, was 51.9%. Similar studies have reported widely varying infection rates, depending on their accounting for all infections or severe infections only [18,19,23,25]. Other comparative studies, done mostly in the 2000s, looked at infectious death rates and found that older transplant patients tended to do significantly worse than younger recipients [17,26], with deaths being more often due to opportunistic agents. The incidence of malignancy at one year and 10 years post transplant was 3.8% and 33.3%, respectively, in our study. This is comparable to prior studies, where the incidence of malignancy increases with time after transplant [19,27].

Post-transplant incidence of rejection in our study was 9.6%, with no differences based on donor status. Huang et al., in 2010, compared outcomes in three cohorts of ages 60-69, 70-79, and above 80 years and reported no difference in rates of rejection (4.7% versus 4.2% versus 4.5%, respectively) between the three groups [22]. Similarly, Boesmueller and colleagues found a comparable rate of acute rejection in their comparison groups of recipients older than 70 years with younger recipients (15.8% versus 17.8%) [18], as did Bharagava et al., who looked at kidney transplant recipients older than 60 years with younger recipients (11.3% versus 10.2%) [24]. Owing to increased infectious complications noted in earlier studies, centers may opt to wean immunosuppression in elderly recipients, and this may lead to variable incidences of reported rejection in different study cohorts [18,22,24]. In our study group, the only predictive factor for early graft loss based on recipient comorbidity was a history of a cerebrovascular event and CAD. DGF and return to the operating room were the only perioperative factors contributing to early graft loss.

There are several limitations to the study, which must be addressed. First, the retrospective study design has well-known limitations. Second, this was a single-center study, which allowed consistency in the way patients were evaluated, waitlisted, transplanted, and managed; however, generalizability is limited and the present findings may not be applicable to other institutions or patient populations. The small sample size introduces the possibility of a Type II error. There is a need for other similar studies that look at outcomes in kidney transplant patients solely above 70 years of age in order to confirm our results are not limited to our institution. Owing to the retrospective nature of the study, post-transplant changes in functional independence were not able to be known. Lastly, there are other potential factors such as frailty syndrome, dementia, delirium, and malnutrition that may impact graft survival, which were not accounted for in this analysis.

## Conclusions

Our study reports acceptable short-term outcomes in a sample of kidney transplant recipients over the age of 70 years. Graft survival is similar to rates seen in studies that looked at younger cohorts but the decline in this rate over time is steeper in the older age group and may possibly be due to decreased patient survival. Thus, the expected transplant advantage in terms of long-term patient survival is not realized in elderly patients included in our study. Considering the shortage of kidney donors, anticipated life expectancy has to be equated with expected outcomes. More studies on quality-of-life benefits and post-transplant changes in functional independence are needed to validate appropriate resource utilization among older recipients.

## References

[1] (2022). 2017 National Population Projections Tables: Main Series. Main Series.

[2] (2022). An Aging Nation: Projected Number of Children and Older Adults. https://www.census.gov/library/visualizations/2018/comm/historic-first.html.

[3] Kovesdy CP (2022). Epidemiology of chronic kidney disease: an update 2022. Kidney Int Suppl (2011).

[4] (2021). OPTN/SRTR 2019 annual data report: introduction. Am J Transplant.

[5] Hernández D, Alonso-Titos J, Armas-Padrón AM (2018). Mortality in elderly waiting-list patients versus age-matched kidney transplant recipients: where is the risk?. Kidney Blood Press Res.

[6] Huang E, Segev DL, Rabb H (2009). Kidney transplantation in the elderly. Semin Nephrol.

[7] So S, Au EH, Lim WH, Lee VW, Wong G (2021). Factors influencing long-term patient and allograft outcomes in elderly kidney transplant recipients. Kidney Int Rep.

[8] Dempster NJ, Ceresa CD, Aitken E, Kingsmore D (2013). Outcomes following renal transplantation in older people: a retrospective cohort study. BMC Geriatr.

[9] Knoll GA (2013). Kidney transplantation in the older adult. Am J Kidney Dis.

[10] Shlipak MG, Katz R, Kestenbaum B (2009). Rate of kidney function decline in older adults: a comparison using creatinine and cystatin C. Am J Nephrol.

[11] (2023). Organ Procurement and Transplantation Network. https://portal.unos.org/.

[12] (2023). Kidney Donor Profile Index (KDPI) Guide for Clinicians. https://optn.transplant.hrsa.gov/professionals/by-topic/guidance/kidney-donor-profile-index-kdpi-guide-for-clinicians/.

[13] Wolfe RA, Ashby VB, Milford EL (1999). Comparison of mortality in all patients on dialysis, patients on dialysis awaiting transplantation, and recipients of a first cadaveric transplant. N Engl J Med.

[14] Rao PS, Merion RM, Ashby VB, Port FK, Wolfe RA, Kayler LK (2007). Renal transplantation in elderly patients older than 70 years of age: results from the Scientific Registry of Transplant Recipients. Transplantation.

[15] Schold J, Srinivas TR, Sehgal AR, Meier-Kriesche HU (2009). Half of kidney transplant candidates who are older than 60 years now placed on the waiting list will die before receiving a deceased-donor transplant. Clin J Am Soc Nephrol.

[16] Lentine KL, Smith JM, Hart A (2022). OPTN/SRTR 2020 annual data report: kidney. Am J Transplant.

[17] Calabuig AS, Martínez EG, Berga JK (2018). Kidney transplantation in recipients older than 70 years old: a good option for our patients. Transplantation.

[18] Boesmueller C, Biebl M, Scheidl S (2011). Long-term outcome in kidney transplant recipients over 70 years in the Eurotransplant Senior Kidney Transplant Program: a single center experience. Transplantation.

[19] Lemoine M, Titeca Beauport D, Lobbedez T (2019). Risk factors for early graft failure and death after kidney transplantation in recipients older than 70 years. Kidney Int Rep.

[20] Frei U, Noeldeke J, Machold-Fabrizii V (2008). Prospective age-matching in elderly kidney transplant recipients--a 5-year analysis of the Eurotransplant Senior Program. Am J Transplant.

[21] Fabrizii V, Winkelmayer WC, Klauser R (2004). Patient and graft survival in older kidney transplant recipients: does age matter?. J Am Soc Nephrol.

[22] Huang E, Poommipanit N, Sampaio MS (2010). Intermediate-term outcomes associated with kidney transplantation in recipients 80 years and older: an analysis of the OPTN/UNOS database. Transplantation.

[23] Doucet BP, Cho Y, Campbell SB, Johnson DW, Hawley CM, Teixeira-Pinto AR, Isbel NM (2021). Kidney transplant outcomes in elderly recipients: an Australia and New Zealand dialysis and transplant (ANZDATA) registry study. Transplant Proc.

[24] Bhargava V, Meena P, Agrawaal K (2021). Outcomes of kidney transplantation in the elderly recipients. Indian J Nephrol.

[25] Lim JH, Lee GY, Jeon Y (2022). Elderly kidney transplant recipients have favorable outcomes but increased infection-related mortality. Kidney Res Clin Pract.

[26] Danovitch GM, Gill J, Bunnapradist S (2007). Immunosuppression of the elderly kidney transplant recipient. Transplantation.

[27] Lemoine M, Guerrot D, Bertrand D (2018). Focusing on kidney transplantation in the elderly (Article in French). Nephrol Ther.

